# Carmofur Exhibits Antimicrobial Activity Against *Streptococcus pneumoniae*

**DOI:** 10.3390/antibiotics14030231

**Published:** 2025-02-25

**Authors:** Wenting Lyu, Yuqing Zhang, Zhen Zhang, Hao Lu

**Affiliations:** 1 College of Pharmacy, Heze University, Heze 274000, China; lywl1@student.unimelb.edu.au (W.L.); yuqinzhang9@student.unimelb.edu.au (Y.Z.); 2 Faculty of Medicine, Dentistry and Health Sciences, The University of Melbourne, Melbourne 3010, Australia; 3 Yangtze Delta Region Institute of Tsinghua University, Jiaxing 314006, China; zhangzhen@tsinghua-zj.edu.cn

**Keywords:** *S. pneumoniae*, multidrug resistant, carmofur, antibacterial, mouse pneumonia

## Abstract

**Background/Objectives:** *Streptococcus pneumoniae* (*S. pneumoniae*) is a major pathogen causing severe infectious diseases, with an escalating issue of antimicrobial resistance that threatens the efficacy of existing antibiotics. Given the challenges in developing traditional antibiotics, drug repurposing strategies offer a novel approach to address the resistance crisis. This study aims to evaluate the antibacterial and anti-biofilm activities of the approved non-antibiotic anticancer drug carmofur against multidrug-resistant *S. pneumoniae*, and investigate the mechanism of action, and assess therapeutic potential in vivo. **Methods/Results:** Antimicrobial tests revealed that carmofur exhibited strong antibacterial activity against multidrug-resistant *S. pneumoniae* strains, with minimum inhibitory concentrations (MICs) ranging from 0.25 to 1 µg/mL. In the biofilm detection experiments, carmofur not only inhibited the formation of biofilms, but also effectively removed biofilms under high concentration conditions. Mechanistic studies showed that carmofur disrupted bacterial membrane permeability and decreased intracellular ATP levels. Molecular docking and dynamics simulation assays indicated that carmofur could stably bind to thymidylate synthase through hydrogen bonding and hydrophobic interactions, thereby exerting antibacterial effects. Meanwhile, carmofur was able to repress the expression of the *thyA* gene at the mRNA level. In a mouse infection model, the carmofur treatment group showed a reduction of approximately two log levels in bacterial load in lung tissue and blood, a significant decrease in the levels of inflammatory cytokines TNF-α and IL-6, and an improvement in survival rate to 60%. **Conclusions:** In summary, carmofur demonstrated significant antibacterial and anti-biofilm activities against multidrug-resistant *S. pneumoniae* and showed good anti-infective effects in vivo, suggesting its potential clinical application as a therapeutic agent against drug-resistant bacteria.

## 1. Introduction

*Streptococcus pneumoniae* (*S. pneumoniae*) is a common inhabitant of the human upper respiratory tract and a major pathogen responsible for severe infectious diseases, such as pneumonia, otitis media, sinusitis, meningitis, and septicemia [1,2]. *S. pneumoniae* is mainly transmitted through respiratory droplets and poses a high risk of infection in children, the elderly and immunocompromised individuals [3,4]. According to the World Health Organization (WHO), *S. pneumoniae* causes approximately 1.5 million deaths annually in children under five, with infection rates significantly higher in low-income countries compared to high-income regions [5]. Data from the Centers for Disease Control and Prevention (CDC) indicate that *S. pneumoniae* is the leading cause of community-acquired pneumonia (CAP), responsible for about 30–50% of community-acquired pneumonia cases in adults [6,7]. Moreover, *S. pneumoniae*-induced meningitis is highly lethal, with an estimated mortality rate of 20% in children and up to 60% in the elderly, even with treatment [8]. Survivors often suffer from neurological damage and hearing impairment, significantly reducing their quality of life. Globally, pneumococcal infections not only pose a serious threat to patient lives, but also impose substantial economic burdens. In the United States, the annual direct medical costs and indirect losses attributed to *S. pneumoniae* infections amount to approximately USD 3 billion, while in Europe, the costs are even higher due to a larger elderly population and associated infection risks [9].

In recent years, the widespread and irrational use of antibiotics has led to a significant increase in *S. pneumoniae* resistance, particularly to penicillins, cephalosporins, and macrolides [10]. The rise in resistance not only complicates clinical treatment, but also severely impacts the efficacy of antimicrobial agents [11]. According to World Health Organization statistics, around 40–60% of *S. pneumoniae* isolates globally exhibit varying levels of resistance, with resistance rates reaching as high as 80% in certain high-resistance regions, such as Southeast Asia and Africa [12]. Macrolides, used as alternative therapies for *S. pneumoniae* infections, are also facing rising resistance rates, with macrolide resistance in the United States and China reaching 25% and 50%, respectively, posing a significant challenge [13]. The issue of pneumococcal resistance presents major challenges for clinical treatment and increases the risk of bacterial infection outbreaks [14]. Vaccination is considered a key preventive measure against *S. pneumoniae* infections and resistance development [15]. Pneumococcal conjugate vaccines (PCV13) and the 23-valent pneumococcal polysaccharide vaccine (PPSV23) have been widely administered, effectively reducing infection rates among children and high-risk populations globally [16]. However, despite the positive impact of vaccines in lowering infection rates, their effect on resistant strains remains uncertain. Some studies have observed a replacement effect, whereby non-vaccine serotype-resistant strains increase in prevalence within vaccinated populations, creating new challenges in resistance control [17]. In this context, developing new antimicrobial strategies and drugs is particularly urgent.

The “repurposing” strategy for non-antibiotic drugs offers a new avenue for the development of anti-*S. pneumoniae* agents [18]. Drug repurposing involves redeveloping approved drugs for different indications, particularly against resistant bacteria. This strategy circumvents traditional antibiotic targets and utilizes novel mechanisms to interfere with bacterial metabolism or physiological processes, achieving inhibitory effects on resistant strains [19]. Repurposing approved non-antibiotic drugs as anti-*S. pneumoniae* agents offers distinct advantages [20]. Toxicological studies and clinical safety evaluations have already been completed, facilitating faster entry into clinical trials and significantly reducing development time and cost. Additionally, the molecular structure, metabolic pathways, and dose–response relationships of these drugs are well-characterized, providing a solid reference for their use at antibacterial doses [21,22]. Moreover, many non-antibiotic drugs exhibit good tissue penetration and broad-spectrum activity, potentially inhibiting various resistant bacteria at low doses and offering new treatment options for patients with multidrug-resistant infections [23,24]. Therefore, exploring approved non-antibiotic drugs as potential therapies for *S. pneumoniae* is a promising strategy.

Carmofur, a fluorouracil-based antimetabolite, was initially developed in the 1980s for cancer treatment, particularly as an adjuvant therapy for colorectal cancer [25,26,27]. Studies have found that carmofur was a rat recombinant acid ceramidase inhibitor with an IC50 of 29 nM. Carmofur was also a protease inhibitor of SARS-CoV-2 main protease (Mpro), fatty acid amide hydrolase (FAAH) and N-acylethanolamine acid amidase (NAAA). Meanwhile, carmofur had anticancer, anti-inflammatory and anti-virus activities, and could be used for the study of COVID-19 and acute lung injury (ALI) [28,29,30]. However, carmofur has not been reported in terms of its antimicrobial potential against multi-resistant *S. pneumoniae*. In this study, we elucidated the antibacterial and anti-biofilm effects of carmofur against *S. pneumoniae* in detail, and further analyzed its antibacterial mechanism by using computational biology. Meanwhile, we preliminarily investigated the therapeutic effects of carmofur against *S. pneumoniae* infected animals. These data provided strong support for the development of carmofur as a novel anti-*S. pneumoniae* drug, and also offered a new idea and clinical application prospect for the current anti-infection treatment field with rising drug resistance.

## 2. Results

### 2.1. Carmofur Exhibited Potent Antibacterial Activity Against S. pneumoniae

**Figure 1:**
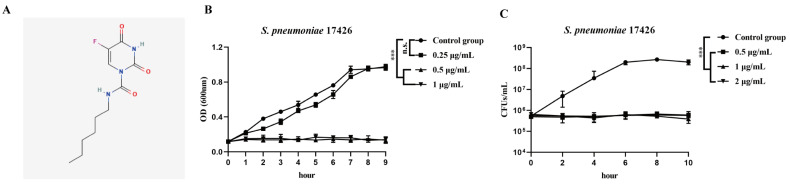
Effects of different concentrations of carmofur on the growth of *S. pneumoniae* strain 17426. (**A**) The 2D structural formula of carmofur; (**B**) bacterial growth curves based on optical density (OD 600 nm) over time. Carmofur concentrations were set at 0.25 µg/mL, 0.5 µg/mL, and 1 µg/mL. Untreated control was used as a reference; (**C**) changes in colony-forming units (CFUs/mL) over time in carmofur-treated groups. Concentrations of 0.5 µg/mL, 1 µg/mL, and 2 µg/mL were tested. Each group consisted of three biological replicates. Data are presented as mean ± standard deviation (Mean ± SD). *** *p* < 0.001, and n.s. indicates not significant. The statistical analysis at the end of the experiment was performed via Student’s *t* test.

The structure of carmofur is shown in Figure 1A. To assess the antibacterial activity of carmofur, we measured the minimum inhibitory concentration (MIC) against 13 strains of *S. aureus* and *S. pneumoniae*, including both drug-resistant and non-resistant strains. Among the *S. pneumoniae* isolates, all strains exhibited resistance to multiple antibiotics, including erythromycin, clindamycin, azithromycin, tetracycline, and trimethoprim-sulfamethoxazole, with some strains also resistant to penicillin and gentamicin, reflecting broad multidrug resistance (Table 1). Specifically, the MICs of carmofur against *S. pneumoniae* ranged from 0.25 to 1 µg/mL, showing variability in susceptibility among strains (Table 1). Similarly, for *S. aureus* strains, carmofur displayed an MIC of 0.25 µg/mL for the standard non-resistant ATCC25923 strain and 0.5 µg/mL for the MRSA strain ATCC43300, indicating strong antibacterial activity against different bacterial species (Table 1). Overall, carmofur demonstrated considerable inhibitory efficacy against multidrug-resistant *S. pneumoniae.*

To further evaluate the antibacterial efficacy of carmofur, we examined the effects on growth and bactericidal activity against a clinical multidrug-resistant *S. pneumoniae* strain 17426. Figure 1B shows the bacterial growth curves based on optical density (OD) measurements at 600 nm. The control group exhibited typical exponential growth, with OD values steadily increasing and peaking at around 1.0 by 9 h. In the carmofur-treated groups, bacterial growth was significantly inhibited in a concentration-dependent manner, with minimal growth observed at concentrations of 0.5 µg/mL and 1 µg/mL, indicating substantial growth suppression at these doses. However, sub-MIC concentrations did not demonstrate inhibitory effects (Figure 1B). Given that sub-MIC concentrations do not inhibit bacterial growth, we chose MIC, 2-fold MIC, and 4-fold MICs for testing in the time killed curves. Figure 1B illustrates the impact of carmofur treatment on the number of culturable bacteria (CFUs/mL). In the control group, CFU counts increased progressively, reaching over 10^8^ CFUs/mL by 6 h, after which a slight plateau was observed (Figure 1C). In contrast, the carmofur-treated groups (0.5 µg/mL, 1 µg/mL, and 2 µg/mL) maintained the initial bacterial count throughout the experiment, with no significant increase over time, indicating strong bacteriostatic effects at these concentrations (Figure 1C). However, there was a significant difference in the bacterial load in the carmofur treated group as compared to the control group at 10 h (Figure 1C). In conclusion, these data suggested that carmofur had antimicrobial effects against multi-resistant *S. pneumoniae*.

### 2.2. Inhibition and Eradication of S. pneumoniae Biofilm by Carmofur

**Figure 2:**
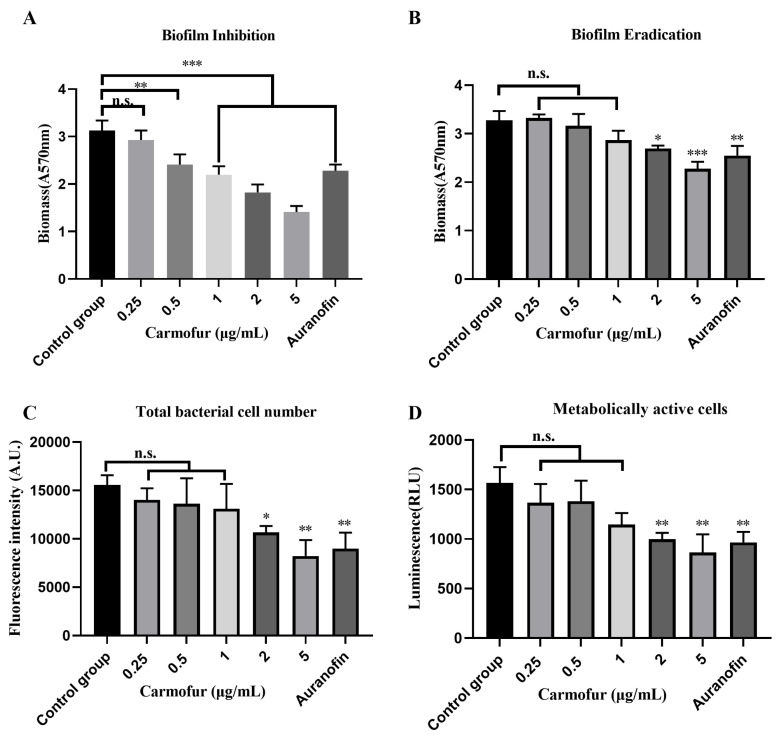
Anti-biofilm effects of carmofur (0.25, 0.5, 1, 2 and 5 µg/mL) against *S. pneumoniae*. Auranofin (MIC = 0.5 µg/mL), a known reported drug, served as a positive control. (**A**) Inhibition of biofilm formation at varying concentrations of carmofur. (**B**) Eradication effect on pre-formed biofilms. (**C**) Total bacterial count in mature biofilms, evaluated with acridine orange fluorescence probe at different carmofur concentrations. (**D**) Reduction in metabolically active cells in mature biofilms, assessed by ATP levels. Each group consisted of three biological replicates. Data are presented as Mean ± SD. Significance levels are indicated as follows: *, *p* < 0.05; **, *p* < 0.01; ***, *p* < 0.001; ‘n.s.’ indicates no significant difference. Statistical analyses were performed via one-way ANOVA.

Biofilms, protective structures formed by bacteria on host tissues and medical device surfaces, significantly enhance bacterial tolerance to antibiotics [31]. In a 96-well plate assay, we examined the antibiofilm potential of carmofur on different biofilm components using various methods (quantification of biofilm biomass using crystal violet; quantification of total bacterial cell number using acridine orange; and quantification of metabolically active cells within the biofilm using ATP levels). In biofilm inhibition experiments, the control group exhibited the highest biofilm biomass. As carmofur concentration increased (0.25, 0.5, 1, 2 and 5 µg/mL), biofilm biomass progressively decreased (Figure 2A). In particular, the 1, 2 and 5 µg/mL group showed a significant reduction in biofilm biomass (*p* < 0.001), while the 0.5 µg/mL group also demonstrated substantial inhibition (*p* < 0.01) (Figure 2A). However, the 0.25 µg/mL group did not show a significant difference compared to the control, indicating a dose-dependent inhibition effect (Figure 2A). For established biofilms, carmofur’s eradication effect was limited, with the 0.25, 0.5 and 1 µg/mL treatment groups showing no significant difference from the control group (Figure 2B). Only the 2 and 5 µg/mL group displayed a reduction trend (*p* < 0.05 and *p* < 0.001), suggesting that carmofur at higher concentrations possessed some capacity for eradicating pre-formed biofilms (Figure 2B). In measurements of total bacterial counts within biofilms, fluorescence intensity revealed a concentration-dependent reduction in bacterial numbers. Consistently, the fluorescence intensity in the 2 and 5 µg/mL group was significantly lower than that of the control group (*p* < 0.05 and *p* < 0.01), indicating that high concentrations of carmofur effectively reduced the total bacterial count in biofilms (Figure 2C). The 0.25, 0.5 and 1 µg/mL groups did not differ significantly from the control (Figure 2C). Additionally, in the evaluation of metabolically active cells within the biofilm, luminescence intensity showed a significant decrease in the 2 and 5 µg/mL group (*p* < 0.01), while the 0.25, 0.5 and 1 µg/mL groups were not significantly different from the control, further indicating that high-concentration carmofur had a marked inhibitory effect on metabolically active cells within the biofilm (Figure 2D). Collectively, these findings suggested that carmofur could serve as a potential antibiofilm agent, addressing biofilm-related resistance issues in bacterial infections.

### 2.3. Carmofur Alters Bacterial Membrane Permeability and Affects Bacterial Energy Metabolism

**Figure 3:**
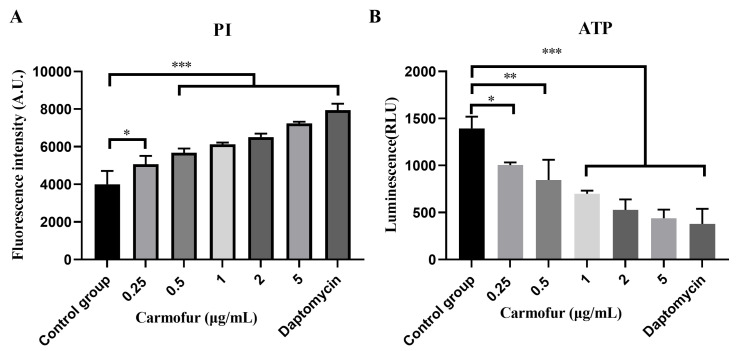
Antibacterial mechanism of carmofur. (**A**) PI uptake in *S. pneumoniae*. (**B**) Analysis of intracellular ATP levels in *S. pneumoniae* treated with various concentrations of carmofur. Daptomycin (8 µg/mL) served as a positive control. Each group consisted of three biological replicates. Data are presented as mean ± SD. Significance levels are indicated as follows: *, *p* < 0.05; **, *p* < 0.01; ***, *p* < 0.001. The statistical analysis at the end of the experiment was performed via one-way ANOVA.

Although carmofur was primarily an anticancer drug [32], its antibacterial activity may involve altering bacterial membrane permeability, allowing the drug to penetrate the bacterial cell and interfere with its metabolic processes, thus inhibiting bacterial growth and activity. To verify this hypothesis, we evaluated the effects of carmofur by measuring changes in propidium iodide (PI) fluorescence intensity and ATP levels. A membrane-active antibiotic, daptomycin, served as a positive control. PI is a membrane-impermeable red fluorescent dye that stains DNA. PI is usually excluded from living cells and can penetrate cells with damaged membranes. The results showed that as carmofur concentration increased, bacterial PI fluorescence intensity also gradually increased, especially at high concentrations (0.5–5 µg/mL), with significant differences compared to the control group (*p* < 0.001) (Figure 3A). PI is a dye that can only penetrate cells with damaged membranes [33], indicating that carmofur compromised membrane integrity, increasing permeability and allowing dye entry into bacterial cells. The high concentration of the control drug daptomycin resulted in a significant increase in fluorescence intensity, which represented the reliability of the experimental results (Figure 3A). Furthermore, ATP content analysis demonstrated a significant and concentration-dependent decrease in bacterial ATP levels following carmofur treatment, with all tested concentrations showing significant differences compared to the control (Figure 3B). ATP was a key molecule in bacterial metabolism, and its reduction indicated impaired bacterial viability [34], likely due to carmofur compromising the cell membrane structure, which reduced bacterial ability to acquire energy and nutrients, thus impacting metabolic processes and inhibiting bacterial replication.

### 2.4. Carmofur Binded to Bacterial Thymidylate Synthase Protein via Hydrogen Bonding and Hydrophobic Interactions

**Figure 4:**
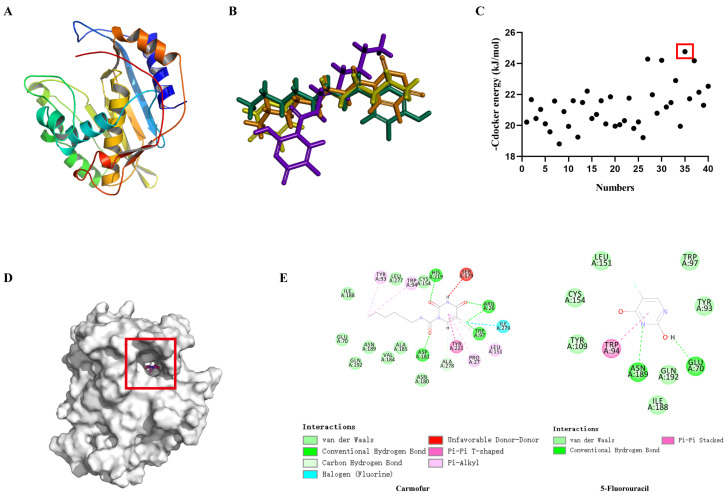
Molecular docking and interaction analysis of carmofur with target protein. (**A**) Three-dimensional structure of the target protein, comprising typical α-helices and β-sheets; (**B**) superimposed results of carmofur’s four primary binding conformations, showing its spatial compatibility; (**C**) distribution of binding energies (Cdocker energy) across different conformations, with the lowest binding energy (around −25 kJ/mol) marked in the red box as the optimal conformation; (**D**) detailed interaction analysis of carmofur and 5-fluorouracil within the binding pocket; (**E**) two-dimensional spatial binding mode of carmofur in the binding pocket, showing multiple interactions.

As a prodrug of 5-fluorouracil (5-FU), we hypothesized that carmofur may also target thymidylate synthase of *S. pneumoniae*. To further investigate the antibacterial mechanism of carmofur, we conducted computational studies to analyze the interaction between carmofur and the thymidylate synthase protein. Since the crystal structure of *S. pneumoniae* thymidylate synthase (*thyA*) has not yet been resolved, we used the protein structure from the AlphaFold2 database as the study target. Examination of the three-dimensional structure revealed that the target protein contains typical α-helix and β-sheet structures, providing a stable framework for carmofur binding (Figure 4A). Four primary conformations of carmofur were identified, as shown in the figure (Figure 4B). Binding energy analysis indicated the optimal binding conformation, with a binding energy of −25 kJ/mol, which is significantly lower than other conformations, suggesting this conformation had the highest stability and binding efficiency (Figure 4C). Based on this, the optimal binding conformation was selected for further mechanistic analysis. 5-FU was used as a control drug. The overall view of the binding site showed that carmofur and 5-FU were simultaneously bound in the same hydrophobic pocket on the protein surface (Figure 4D). Detailed interaction analysis revealed that carmofur formed stable intermolecular interactions in the binding pocket, including van der Waals forces, hydrogen bonding, hydrophobic interactions, and π-π stacking (Figure 4E). Specifically, carmofur formed hydrogen bonds with TYR93 and SER179, and hydrophobic interactions with LEU151 and TYR221, further strengthening binding stability (Figure 4E). Additionally, carmofur enhanced binding efficiency through multi-point interactions with ARG26 and HIS219, creating a tight interaction network (Figure 4E). In contrast, 5-FU only interacted with TRP94, ASN189, and GLU70 of the protein (Figure 4E). In short, the charge distribution and shape of the binding region were highly compatible with carmofur, forming an environment conducive to stable binding on the pocket surface.

**Figure 5:**
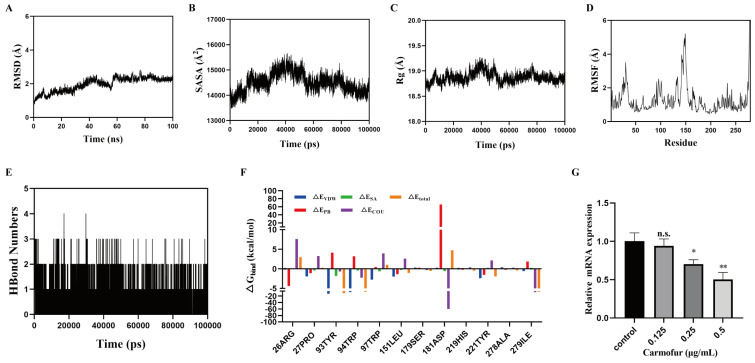
The effects of carmofur on thymidylate synthase. (**A**) The root mean square deviation (RMSD) as a function of time, indicating that the system gradually reached stability during the molecular dynamics simulation; (**B**) the solvent-accessible surface area (SASA) over time, reflecting fluctuations in protein solvent accessibility; (**C**) the radius of gyration (Rg) as a function of time, demonstrating the overall compactness of the protein conformation; (**D**) the root mean square fluctuation (RMSF) analysis, revealing the conformational flexibility of different protein residues; (**E**) the number of hydrogen bonds over time, illustrating the dynamic characteristics of hydrogen bonding interactions within the system; (**F**) the binding free energy decomposition results, showing the energetic contributions of different residues to ligand binding; (**G**) the effect of carmofur on relative mRNA expression levels. Data are presented as mean ± standard deviation (mean ± SD); mean ± SD was repeated three times. * *p* < 0.05, ** *p* < 0.01; n.s. indicates no significant difference. The statistical analysis at the end of the experiment was performed Via Student’s *t* test.

To further explore how carmofur affected the protein, molecular dynamics simulations were used to analyze the interaction between carmofur and thymidylate synthase. The root mean square deviation (RMSD) was a reliable indicator for assessing the conformational stability of proteins and ligands, as well as the deviation of atomic positions from their initial coordinates. A smaller deviation indicated greater conformational stability. Therefore, RMSD was used to evaluate the equilibrium of the simulation system. As shown in Figure 5A, the *thyA*-carmofur complex system reached equilibrium after 60 ns, with fluctuations around 2.5 Å and 2.4 Å. This suggested that the binding of the small molecule carmofur to the *thyA* target protein exhibited high stability. Further analysis revealed that the solvent-accessible surface area (SASA) and radius of gyration (Rg) of the *thyA*-carmofur complex exhibited slight fluctuations during the simulation, indicating that the binding of the small molecule induced conformational changes in the target protein (Figure 5B,C). The root mean square fluctuation (RMSF) reflected the flexibility of amino acid residues within the protein. As shown in Figure 5D, the RMSF values of the *thyA*-carmofur complex system were relatively low (mostly below 3 Å), suggesting low flexibility and high structural stability. Hydrogen bonds played a crucial role in ligand–protein interactions. For the period during the simulation, the number of hydrogen bonds formed between carmofur and the target protein is shown in Figure 5E. The number of hydrogen bonds in the *thyA*-carmofur complex system ranged from 0 to 4, with an average of approximately 3 hydrogen bonds in most cases. This indicated strong hydrogen bonding interactions within the *thyA*-carmofur complex. Subsequently, key amino acid residues contributing to the binding of the small molecule in the *thyA*-carmofur complex system were further analyzed. The results revealed that residues ARG26, TYR93, TRP94, ASP181, and ILE279 exhibited high energy contributions (Figure 5F), highlighting their crucial roles in the binding process. These findings demonstrated the binding stability of the *thyA*-carmofur complex. Furthermore, the effect of carmofur on the transcriptional level of the *thyA* gene was examined. As shown in Figure 5G, *thyA* gene expression levels exhibited a dose-dependent decreasing trend with increasing concentrations of carmofur. The inhibition was the most significant at 0.5 µg/mL (*p* < 0.01), whereas no significant difference was observed at the low concentration of 0.125 µg/mL. In conclusion, molecular dynamics simulations confirmed the stability of the system throughout the simulation period. Free energy decomposition analysis identified key contributing residues, and experimental results validated the inhibitory effect of carmofur on gene expression within a certain concentration range. These findings provided important insights into the potential pharmacological mechanisms of carmofur.

### 2.5. Evaluation of the Therapeutic Efficacy of Carmofur in a Mouse Model of Streptococcus pneumoniae Infection

**Figure 6:**
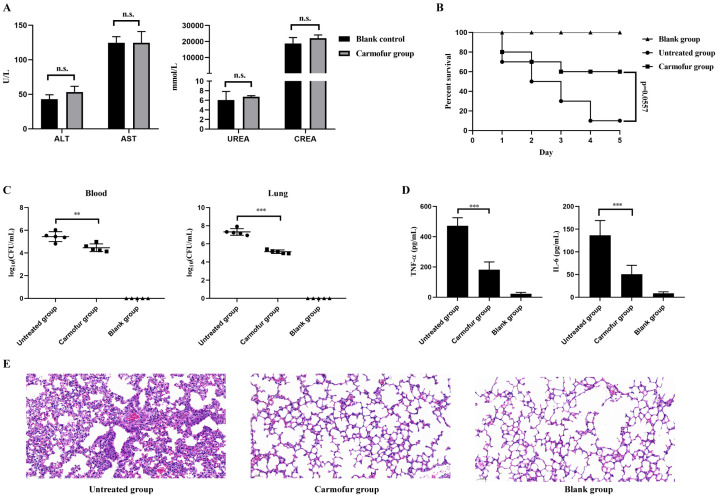
Therapeutic efficacy of carmofur in mice infected with *S. pneumoniae*. (**A**) Results of blood biochemical indexes to evaluate the effects of carmofur on liver and kidney functions in mice. (**B**) Survival rate of mice treated with carmofur 1 h post-infection with an inoculum dose of 5 × 10^8^ CFU. (**C**) Bacterial load in blood and lung tissues of mice. (**D**) Levels of TNF-α and IL-6 in mouse serum. (**E**) Histopathological changes in lung tissue post-treatment. Data are presented as mean ± standard deviation (Mean ± SD). ** *p* < 0.01, *** *p* < 0.001; n.s. indicates no significant difference. The statistical analysis at the end of the experiment was performed Via Student’s *t* test.

To assess the in vivo antibacterial efficacy of carmofur, this study utilized a mouse pneumonia model infected with *S. pneumoniae* to evaluate carmofur’s therapeutic effects. Given that carmofur was commonly used as an anticancer drug, here we referred to the relevant literature and chose one-tenth of the LD_50_ dose (96 mg/kg) in mice resulting from intraperitoneal injection for the treatment study. Firstly, this study was also conducted to assess the potential toxic effects of the same dose of carmofur in mice by testing their blood biochemical parameters. The levels of alanine aminotransferase (ALT) and azelaic aminotransferase (AST) were measured to assess liver function, while the levels of urea (UREA) and creatinine (CREA) were determined to assess renal function. The results showed that there was no significant difference (*p* > 0.05) in the ALT levels of mice in the carmofur-treated group compared to the blank control group, indicating that carmofur had less effect on hepatocellular injury (Figure 6A). In addition, AST levels were also not significantly different between the two groups (*p* > 0.05), further indicating that carmofur did not cause significant liver injury (Figure 6A). In terms of renal function assessment, the levels of UREA and CREA in the carmofur-treated group were not significantly different from those of the blank control group (*p* > 0.05), indicating that carmofur did not cause significant effects on renal function in mice (Figure 6A). Thus, carmofur did not cause significant hepatorenal toxicity under the present experimental conditions, providing supportive evidence for its safety assessment in further studies. Subsequently, carmofur demonstrated significant anti-infective and protective effects in this infection model. As shown in Figure 6B, the following survival rates across different treatment groups were observed: the blank group maintained a 100% survival rate over 5 days post-infection, whereas the untreated group experienced a progressive decline in survival, reaching 10%. In contrast, the carmofur-treated group showed extended survival time, with a final survival rate of 60%, though still lower than the blank group. Further analysis of bacterial load post-infection (Figure 6C) revealed that the colony-forming units (CFUs) in blood and lung tissues of the carmofur-treated group were significantly lower than those in the untreated group, reaching significance levels of *p* < 0.01 and *p* < 0.001, respectively. This indicated that carmofur effectively suppressed bacterial proliferation in vivo, reducing bacterial counts at the infection sites. Regarding inflammatory response (Figure 6D), the untreated group exhibited significantly elevated levels of pro-inflammatory cytokines TNF-α and IL-6, reflecting a strong infection-induced inflammatory response. In contrast, the carmofur-treated group showed a significant reduction in TNF-α and IL-6 expression levels (*p* < 0.001), almost approaching the levels observed in the blank group. This suggested that carmofur could mitigate tissue damage caused by infection by suppressing excessive inflammatory responses. Histopathological examination of lung tissue sections (Figure 6E) further supported these findings. Lung tissue from the untreated group displayed severe pathological damage, including marked interstitial edema and extensive inflammatory cell infiltration. In comparison, lung tissue from the carmofur-treated group exhibited significantly reduced pathological damage, with fewer inflammatory cells and a more preserved tissue structure, closely resembling that of the blank group. In summary, carmofur in this model not only significantly reduced bacterial load but also effectively suppressed inflammatory responses and alleviated pathological lung tissue damage, demonstrating its potential anti-infective protective effects.

## 3. Discussion

As a derivative of 5-fluorouracil, carmofur has been highlighted in recent studies for its unique pharmacological properties and potential in antimicrobial research. Unlike similar drugs such as 5-fluorouracil or tegafur, carmofur exhibited higher lipophilicity, allowing better cellular penetration and enhanced bioavailability [35]. Studies have shown its significant activity against multidrug-resistant pathogens, such as methicillin-resistant *S. aureus* (MRSA), including its ability to disrupt biofilms [36,37]. High-throughput screening studies further supported its potential for drug repurposing, particularly in infection models like *Pseudomonas aeruginosa*-related cystic fibrosis [38]. Additionally, carmofur may demonstrate a unique mechanism by inhibiting acid ceramidase, which was not commonly targeted by its counterparts, offering novel insights into antimicrobial mechanisms [39]. With better metabolic stability, lower cytotoxicity, and broader antimicrobial applications, carmofur stood out as a promising candidate for further investigation. This study further systematically investigated the antibacterial effects of carmofur against *S*. *pneumoniae*, focusing specifically on its inhibition and eradication of biofilms. Carmofur, an approved antimetabolite originally developed for cancer therapy, inhibits tumor cell proliferation primarily by blocking DNA synthesis through thymidylate synthase inhibition [40,41]. However, recent studies have highlighted carmofur’s potential in the anti-infective field [42]. References have shown that carmofur possesses inhibitory activity under certain conditions against both Gram-positive and Gram-negative bacteria, such as *S*. *aureus* and *P. aeruginosa* [38,43]. However, previous research has mainly focused on bacterial growth inhibition assays, lacking in-depth analysis of its mechanism and validation through in vivo infection models. Compared to previous studies, this research not only confirmed the inhibitory effects of carmofur on *S*. *pneumoniae*, but also systematically revealed its anti-biofilm effects against multidrug-resistant *S*. *pneumoniae* in vitro. Our experiments demonstrated that carmofur could effectively inhibit *S*. *pneumoniae* growth at lower concentrations, while at higher concentrations, it significantly eradicated established biofilms. Previous studies on the antibacterial activity of carmofur have been relatively scattered and lack systematic data supporting its potential in biofilm inhibition, particularly in terms of biofilm metabolic activity and bacterial counts [44]. Our findings further validated carmofur’s antibacterial spectrum and its unique mechanism. Prior research has highlighted that the “repurposing” of non-antibiotic drugs is significant in addressing infections caused by drug-resistant bacteria [45]. However, most drugs fail to effectively inhibit bacterial growth and eradicate biofilms simultaneously [46]. Unlike traditional antibiotics, which often showed limited efficacy against biofilm-associated infections, carmofur exhibited notable biofilm suppression effects at higher concentrations, supporting its clinical applicability for anti-biofilm treatment.

As an anticancer drug, carmofur targeted thymidylate synthase, an enzyme closely linked to *S. pneumoniae*’s cell metabolism [47]. Previous study have shown that thymidylate synthase (*thyA*) is essential for bacterial metabolism and virulence [48]. The metabolite of A, 5-FU, an inhibitor of thymidylate synthase, has even been reported to inhibit thymidine synthesis, leading to thymidine-free death and lethal DNA damage in *Streptococcus suis* [49]. Molecular docking in this study elucidated the mechanism of carmofur by demonstrating stable binding with the thymidylate synthase protein in *S. pneumoniae*, thereby inhibiting bacterial proliferation. Thymidylate synthase was an indispensable enzyme in bacterial nucleic acid metabolism, and its primary function was to catalyze the conversion of deoxyuridylic acid (dUMP) to deoxythymidylate (dTMP), which was a key precursor required for DNA synthesis [50,51]. Antimicrobial drugs targeted the bacterial thymidylate synthase, which affected DNA synthesis and thus hindered bacterial growth and proliferation [49]. Carmofur binds to the enzyme’s active site through hydrogen bonding and hydrophobic interactions, which may affect the enzyme activity to some extent. This molecular insight provided support for carmofur’s antibacterial mechanism. In addition, carmofur could be metabolized in vivo to 5-FU (5-fluorouracil), which subsequently exerted antitumor effects by inhibiting thymidylate synthase and blocking DNA synthesis [35]. Subsequently, 5-FU exerted antitumor effects by blocking DNA synthesis through inhibition of thymidylate synthase. 5-FU exhibited a similar mechanism of action in bacteria, including inhibition of thymidylate synthase through metabolism to produce FdUMP, which hindered dTMP synthesis and thus affects DNA replication [52]. At the same time, 5-FU could be doped into the bacterial RNA, leading to erroneous protein synthesis and interfering with bacterial physiological functions [49]. It has been shown that 5-FU has an inhibitory effect on some bacteria, but there is a lack of clear evidence on whether carmofur is metabolized to 5-FU in bacteria, and the related mechanism needs to be further explored. However, due to the distinct structural and metabolic differences between bacterial and cancer cells [53,54], carmofur exhibited additional mechanisms in bacteria, such as altering cell membrane permeability and disrupting bacterial energy metabolism. Our experimental results indicated that carmofur treatment significantly increased *S. pneumoniae* membrane permeability and reduced intracellular ATP levels, suggesting that carmofur exerted its antimicrobial action through multiple pathways.

In the in vivo mouse model of *S. pneumoniae* infection, we further assessed carmofur’s antibacterial effects and observed significantly increased survival rates and markedly reduced pro-inflammatory cytokine levels (TNF-α and IL-6). This indicated that carmofur effectively suppressed bacterial proliferation and alleviates inflammatory responses at the infection site. This contrasted sharply with many traditional antibiotics, such as cephalosporins and macrolides, which showed poor performance in drug-resistant bacterial infection models and were often ineffective in suppressing inflammatory responses [55]. Previous studies have reported the half-maximum cytotoxic concentrations of carmofur on Vero cells and IB3-1 cells to be 34.4 µg/mL and 207.6 µg/mL, respectively [28,38]. In contrast, the MIC50 of carmofur against *S. pneumoniae* in the present study was 0.5 µg/mL, and the calculated selectivity indices (SIs) of A were 68.8 and 415.2, respectively, which suggested that its safety was in the acceptable range. In addition, the biggest difference that distinguishes antimicrobial therapy from antitumor therapy is that it does not require long-term medication. Nevertheless, given the high toxicity of antitumor drugs, the safety of the dose of carmofur still needs to be considered for future use in antimicrobial therapy studies. Here, despite carmofur’s promising effects against *S. pneumoniae* in vitro and in vivo, certain limitations exist regarding its clinical application. Firstly, as an anticancer drug, the potential toxicity of high-dose carmofur needs further evaluation, especially regarding its long-term safety. Although previous studies indicate that the median lethal dose (LD_50_) of carmofur is relatively high in mice when administered orally, subcutaneously, or intraperitoneally (268, 260, and 93 mg/kg, respectively), the potential side effects of carmofur in infection treatments necessitate careful consideration [40]. Secondly, while we observed significant anti-biofilm effects of carmofur at higher concentrations in vitro, its efficacy in clearing mature biofilms at lower concentrations was limited. This suggests that in clinical treatment, carmofur may need to be combined with other drugs to enhance biofilm clearance. Additionally, our study primarily focuses on a *S. pneumoniae* infection model, and further exploration of carmofur’s application against other resistant strains could verify its broad-spectrum antibacterial potential and evaluate its synergistic effects with existing antibiotics.

In brief, the antibacterial and anti-biofilm effects of carmofur suggest that it could be a promising therapeutic strategy against *S. pneumoniae*. With carmofur’s low cost and relatively low toxicity profile, its potential in clinical anti-infective treatment warrants further exploration. This study provides a new example of the application of drug repurposing in the anti-infective field, offering a novel option for treating infections caused by drug-resistant bacteria.

## 4. Materials and Methods

### 4.1. Strains, Growth Conditions, and Preparation of Carmofur

The strains used in this study are listed in Table 1. *S. aureus* ATCC 25923 and ATCC 43300, and *S. pneumoniae* ATCC 49619 were obtained from the American Type Culture Collection (ATCC). Clinical *S. pneumoniae* strains used in this study were isolated and preserved in our laboratory. Tryptic Soy Broth (TSB) and Tryptic Soy Agar (TSA) were purchased from Difco Laboratories, Detroit, Michigan, USA. *S. aureus* was cultured in Mueller–Hinton Broth (MHB, Thermo Fisher Scientific, Waltham, MA, USA) or on MHB agar (MHA) plates at 37 °C. *S. pneumoniae* strains were cultured in TSB or inoculated on TSA (Summus Ltd., Shanghai, China) with the addition of 10% (vol/vol) fetal bovine serum (FBS, Sijiqing Ltd., Shanghai, China) at 37 °C. Carmofur (Catalog No.: HY-B0182, Purity: 99.91%) was purchased from MedChemExpress (MCE, Monmouth Junction, NJ, USA) company, dissolved in dimethyl sulfoxide (DMSO, Sigma-Aldrich, Saint Louis, MO, USA), and filtered through a 0.22 µm syringe filter (Millipore, Burlington, MA, USA) to prepare stock solutions of different concentrations.

### 4.2. Determination of Minimum Inhibitory Concentration (MIC)

The antibacterial activity of carmofur was determined for both resistant and sensitive strains following the reference method for antimicrobial susceptibility testing by the Clinical and Laboratory Standards Institute (CLSI) [56]. In this assay, serial two-fold dilutions of the test compound were prepared, and each dilution was mixed with a bacterial suspension at approximately 5 × 10^5^ CFU/mL. The mixtures were incubated at 37 °C for 18–24 h. A control containing only the culture medium without the test compound was also included, along with a blank control containing only the medium. Turbidity at different drug concentrations was compared to the blank control, and the MIC was defined as the lowest concentration of the compound that visibly inhibited bacterial growth.

### 4.3. Growth Curve Assay

The growth curve assay was performed with minor modifications from a previous study [57]. After overnight activation, *S. pneumoniae* 17426 was diluted 1:100 into fresh TSB medium supplemented with 10% FBS and incubated at 37 °C until it reached the logarithmic growth phase (OD600 of approximately 0.4–0.6). Bacteria were harvested by centrifugation, washed with sterile phosphate-buffered saline (PBS) or fresh medium, resuspended to a 0.5 McFarland standard (equivalent to 1 × 10^8^ CFU/mL), and then further diluted 100-fold in TSB medium. Carmofur was then added to the bacterial cultures at final concentrations of 0, 0.25 µg/mL, 0.5 µg/mL, and 1 µg/mL. The cultures were placed in a shaker incubator at 37 °C with constant shaking at 180 rpm to ensure adequate mixing and growth. OD600 readings were taken every hour over a 9 h period using a microplate spectrophotometer at 600 nm. Growth curves were plotted with time (hours) on the x-axis and OD600 on the y-axis to visualize bacterial growth in each treatment group.

### 4.4. Time-Kill Curve Assay

With slight modifications based on prior study [58], a time-kill curve was established to further assess the bactericidal activity of carmofur at varying concentrations. *S. pneumoniae* 17426 was diluted in broth medium containing 5% FBS to achieve a final bacterial concentration of 5 × 10^5^ CFU/mL. Carmofur was added at final concentrations of 0, 0.5 µg/mL, 1 µg/mL, and 2 µg/mL, and the cultures were incubated at 37 °C. Samples were taken at 2-h intervals, serially diluted, and plated on TSA plates, followed by overnight incubation at 37 °C. Colony counts were performed to establish the time-kill curve, and each experiment was repeated three times.

### 4.5. Assessment of Antibacterial Activity Against Bacterial Biofilms

The anti-biofilm potential of the drugs was assessed in 96-well plates using a variety of methods targeting different components of the biofilm: quantification of biofilm biomass using crystal violet; quantification of total bacterial cell number using acridine orange; and quantification of metabolically active cells within the biofilm using ATP levels. Biofilm formation was performed using a 96-well cell culture plate, following a previous reference [59]. Bacteria were cultured overnight in TSB medium at 37 °C with shaking at 180 rpm. The cultures were then diluted 1:100 in fresh TSB medium containing 10% fetal bovine serum (FBS) and grown until an OD600 of 0.6–0.8 was reached. The bacterial suspension was then further diluted 1:100 in fresh medium and added to the 96-well plate. Fresh medium containing carmofur was subsequently added to achieve final concentrations of 0, 0.25, 0.5, 1, 2 and 5 µg/mL, with fresh medium without carmofur serving as a blank control. The plates were incubated at 37 °C for 24 h, the supernatant was then carefully removed with a pipette to eliminate planktonic cells, and the biofilm was washed three times with PBS buffer. After removing PBS, 200 µL of 95% methanol was added to each well to fix the biofilm for 30 min, followed by the removal of the methanol. Once dried, each well was stained with 200 µL of 0.5% crystal violet for 10 min at room temperature. Excess stain was washed off with sterile PBS buffer, and the OD570 was measured with a microplate reader to quantify the biofilm. Auranofin, a known reported drug, served as a positive control [60].

For biofilm eradication assessment, biofilms were first allowed to form by static incubation in 96-well plates at 37 °C for 24 h. After biofilm formation, varying concentrations of carmofur were added to the wells with pre-formed biofilms. A control group (no drug treatment) was also included. Plates were incubated at 37 °C for another 24 h, washed to remove planktonic cells, and stained with 0.5% crystal violet solution for 10 min. After removing excess stain, ethanol was used to dissolve the stained biofilm. Absorbance was measured at 570 nm, reflecting the residual biofilm amount.

For mature biofilm evaluation, biofilms were cultured as described above, and after 24 h of drug treatment, a 2% acridine orange solution was mixed with the bacterial solution at a 1:100 ratio. Following a 15-min incubation, the biofilms were washed three times with PBS and resuspended in PBS buffer. The absorbance of each well was measured at Ex 485 nm/Em 528 nm using a microplate reader.

To assess viable cells in the mature biofilm, 100 µL of sterile PBS buffer was added to each well to detach bacteria from the biofilm. Then, 100 µL of BacTiter-Glo reagent (BacTiter-Glo Microbial Cell Viability Assay, Promega Corporation, Madison, WI, USA) was added to each well according to the manufacturer’s instructions. After a 5-min incubation, luminescence was measured with a microplate reader.

### 4.6. Bacterial Membrane Permeability Assay

The bacterial membrane permeability assay was performed with slight modifications based on a previous reference [61]. After overnight culture, bacteria were transferred and grown to the mid-log phase, washed with PBS buffer, and resuspended to an OD600 of 0.5. Propidium iodide (PI) was added at a final concentration of 10 nmol/L, along with varying concentrations of carmofur, followed by a 1-h incubation at 37 °C. The control group contained no carmofur. Daptomycin (8 µg/mL) served as a positive control. Excess dye was removed by washing with PBS, and 200 µL of the mixture was added to a black 96-well plate. Fluorescence intensity was measured using a multi-function microplate reader at an excitation wavelength of 535 nm and an emission wavelength of 615 nm.

### 4.7. Intracellular Bacterial ATP Detection

The relative content of intracellular bacterial ATP was measured using the BacTiter-Glo™ Microbial Cell Viability Assay kit (Promega, Madison, WI, USA) [62]. Bacteria grown to the logarithmic phase (OD600 = 0.6–0.8) were washed three times with sterile PBS buffer, resuspended in fresh PBS to an OD600 of 0.5, and treated with different concentrations of carmofur in a 37 °C incubator for 1 h. A PBS control was included, with each experimental and control group prepared in triplicate. Daptomycin (8 µg/mL) served as a positive control. Following incubation, the bacterial suspensions were centrifuged to remove the supernatant containing the drug, and the bacterial pellets were resuspended in a lysozyme-containing buffer and digested at 37 °C for 2 h. The supernatant was collected after centrifugation, and an equal volume (100 µL) of bacterial supernatant was mixed with BacTiter-Glo reagent in the dark and transferred to a black, transparent-bottom 96-well plate for a 5-min incubation. Luminescence values were read using a microplate reader, and the relative content of intracellular ATP was expressed as relative luminescence units (RLU).

### 4.8. Molecular Docking

Specific steps for molecular docking were referenced from previous references with minor modifications [63]. The protein structure of *S. pneumoniae* thymidylate synthase was obtained from the AlphaFold2 database (ID: AF-C1CD36-F1-v4). The protein was processed using the “Prepare Protein” function in the Macromolecules module of Discovery Studio 2019. After processing, the prepared protein was opened in a new window, and the binding site was defined in the Receptor–Ligand Interactions module using the Define and Edit Binding Site function. From the receptor cavities, the optimal binding pocket was selected, and the CDOCKER docking program was executed. Specifically, by means of Blind Docking, the program searched the entire protein surface for potential binding regions and selected the most probable binding sites by means of an energy scoring function (scoring function). Subsequently, a molecular dynamics-based docking approach allowed for the rotation of the ligand in degrees of freedom, generating multiple initial conformations. Ultimately, the scoring function was used in docking to evaluate the energetic merit of ligand–protein binding to determine the optimal binding site. The 3D structure of carmofur and 5-Fluorouracil was obtained from PubChem (PubChem CID: 2577 and 3385), and various conformations were generated using Discovery Studio 2019. Each conformation was docked 10 times, and the conformation with the lowest docking energy (in kcal/mol) was considered the best docking complex.

### 4.9. Molecular Dynamics Simulation

First, 100 ns MD simulations of the complexes were performed using Gromacs 2023. The CHARMM 36 force field parameters were used for the protein and the ligand topology was constructed from the GAFF2 force field parameters [64]. Periodic boundary conditions were used to place the protein–ligand complexes in cubic boxes. The TIP3P water model was used to fill the box with water molecules [65]. Electrostatic interactions were handled using the Particle Mesh Ewald (PME) and Verlet algorithms, respectively. Subsequently, 100,000 steps of isothermal isovolumetric systematic equilibrium and isothermal isobaric systematic equilibrium were performed with a coupling constant of 0.1 ps and a duration of 100 ps simulation. Both van der Waals and Coulomb interactions were calculated using a cutoff value of 1.0 nm. Finally, the system was subjected to molecular dynamics simulations using Gromacs 2023 at a constant temperature (300 K) and constant pressure (1 bar) for a total duration of 100 ns.

### 4.10. Thymidylate Synthase (thyA) Gene Expression Analysis

Bacteria grown to the logarithmic phase (OD600 = 0.6–0.8) were washed three times with sterile PBS buffer, resuspended in fresh PBS to an OD600 of 0.5, and treated with different concentrations of carmofur in a 37 °C incubator for 1 h. Bacterial total RNA extraction was performed using the TRIzol method according to the manufacturer’s instructions. Extracted total RNA was reversed with the HiScript II Q RT Super Mix for qPCR (+gDNA wiper), a reverse transcription kit from Vazyme (Nanjing, China). Fluorescence quantitative PCR was performed using ChamQ SYBR qPRC Master Mix from Vazyme, and the bacterial 16S rRNA gene was selected as an internal reference. It was then immediately placed on a bio-rad cfx96 real-time fluorescence quantitative PCR instrument from the Bio-Rad, Hercules, CA, USA, for quantitative amplification. The program was set as follows: 95 °C for 60 s, 95 °C for 30 s, 60 °C for 60 s, cycling for 40 times, and fluorescence signals were collected during extension. After the reaction was completed, the Bio-Rad CFX Manager 3.0 was used to analyze the solubility curve and the relative expression of genes, and the experimental results were analyzed by the 2^−ΔΔCt^ method to analyze the relative differences in the expression of each group. The primers used are listed in Table 2.

### 4.11. Establishment of Mouse Pneumonia Infection Model

All animal experiments were conducted following animal welfare guidelines and approved by the institutional ethics committee. *S. pneumoniae* 17426 was sub-cultured at a 1:100 ratio in TSB medium and incubated at 37 °C until an OD600 of 0.6 was reached. After centrifugation at 10,000 rpm for 10 min at 4 °C, bacteria were collected and resuspended in sterile PBS buffer (pH 7.4). Thirty 6-week-old female BALB/c mice were randomly divided into three groups. After anesthetizing the mice with ether, 50 µL of *S. pneumoniae* 17426 bacterial suspension (5 × 10^8^ CFU) was administered intranasally to establish a pneumonia model. Blank control and untreated groups were injected with equal amounts of PBS buffer and bacterial suspension, respectively. One hour post-infection, the infected mice were treated with carmofur (10 mg/kg) via intraperitoneal injection, administered every 12 h for three consecutive days. Blank control and untreated groups were injected separately with equal amounts of PBS buffer. Mouse survival was observed for seven consecutive days, and survival curves were plotted. Healthy mice that did not die were finally euthanized. In order to evaluate the toxicity of carmofur to the liver and kidney of mice, mice were randomly divided into blank group and carmofur treatment group (3 mice in each group). Equal doses of carmofur (10 mg/kg) were administered to the extent described above. Blood samples were collected from the periorbital plexus of anesthetized animals after 7 days. Biochemical analysis was performed using an automatic analyzer (chemray 800, Radu Life Sciences, Shenzhen, China). The levels of alanine aminotransferase (ALT), aspartate aminotransferase (AST), urea (UREA) and creatinine (CREA) were analyzed.

Additionally, a second set of mice (five per group) received an intranasal inoculation of *S. pneumoniae* 17426 (1 × 10^8^ CFU) in 50 µL to establish a pneumonia model. Treatment with carmofur or an equivalent dose of PBS buffer as control was performed as described above. At 12 h post-injection, blood was collected from anesthetized mice via cardiac puncture to analyze the effect of carmofur on serum IL-6 and TNF-α levels in infected mice. Inflammatory cytokines were detected by mouse uncoated ELISA kit (Thermo Fisher Scientific, Waltham, MA, USA). Lung homogenates and blood were serially diluted and plated on TSA plates supplemented with 5% fetal bovine serum. The samples were incubated at 37 °C overnight, and bacterial colonies were counted. Lung tissue was fixed in 4% paraformaldehyde for pathological analysis. All mice were disposed of by euthanasia at the end of the experiment.

### 4.12. Statistical Analysis

Statistical analysis of independent samples was performed by Graphpad Prism 8.0. The results were analyzed by Student’s *t*-test or one-way ANOVA, and the data are expressed as mean ± standard deviation (Mean ± SD). * *p* < 0.05 was considered significant, ** *p* < 0.01 was considered highly significant, *** *p* < 0.001 was considered extremely significant, and n.s. represented no significant difference.

## 5. Conclusions

This study explored the potential of the approved anticancer drug carmofur in combating multidrug-resistant *S. pneumoniae*, demonstrating significant antibacterial and antibiofilm activities. Mechanistic studies have indicated that carmofur disrupted bacterial membrane permeability, causing extensive membrane damage and reducing intracellular ATP levels, thereby inhibiting bacterial energy metabolism and proliferation. Molecular docking analysis revealed the binding interactions between carmofur and *S. pneumoniae* thymidylate synthase, showing stable binding via multiple interactions, including hydrogen bonds and hydrophobic forces, which served as a robust molecular basis for its antibacterial efficacy. In vivo studies have demonstrated the efficacy of carmofur in the treatment of *S. pneumoniae*-infected mice. These findings provide critical evidence for the “repurposing” of carmofur as a novel candidate antimicrobial agent against multidrug-resistant *S. pneumoniae*, expanding strategies for antibacterial therapy and offering new directions to address the current antibiotic resistance crisis.

## Figures and Tables

**Table 1:** MICs of carmofur for strains used in the study.

Strains	Source	MIC (µg/mL)						
		Carmofur	ERY	PCN	CLI	AZM	TET	STX
*S. aureus* ATCC 25923	ATCC	0.25	1	0.125	1	2	4	2/32
*S. aureus* ATCC 43300	ATCC	0.5	2	0.25	1	4	4	2/32
*S. pneumoniae* ATCC 49619	ATCC	0.25	0.125	0.5	0.125	0.25	0.5	0.5/2
*S. pneumoniae* 13412	China (Zhe Jiang)	0.5	8	2	8	16	16	8/64
*S. pneumoniae* 14472	China (Zhe Jiang)	0.5	16	16	8	16	32	4/32
*S. pneumoniae* 14466	China (Zhe Jiang)	1	8	4	4	8	16	16/32
*S. pneumoniae* 15131	China (Zhe Jiang)	0.25	32	2	16	8	16	16/32
*S. pneumoniae* 15447	China (Zhe Jiang)	0.25	8	2	16	16	16	8/64
*S. pneumoniae* 16148	China (Zhe Jiang)	0.5	16	4	16	32	32	8/64
*S. pneumoniae* 16227	China (Zhe Jiang)	0.5	16	32	8	8	32	8/64
*S. pneumoniae* 16432	China (Zhe Jiang)	0.5	32	4	8	8	32	16/32
*S. pneumoniae* 17213	China (Zhe Jiang)	1	32	4	8	16	2	16/64
*S. pneumoniae* 17426	China (Zhe Jiang)	0.5	8	32	16	32	32	8/64

ATCC, American Type Culture Collection; ERY, erythromycin; PCN, penicillin; CLI, clindamycin; AZM, azithromycin; TET, tetracycline; SXT, trimethoprim-sulfamethoxazole.

**Table 2:** Sequence of primers used for qPCR analysis.

Gene	Primer	Sequence (5′-3′)
*thyA*	Forward	GGAAGGTTGGGAAGTTCTTC
	Reverse	GGCTTAACAGGGTCATAATCC
16S rRNA	Forward	CATTGTAGCACGTGTGTAGC
	Reverse	AACCTTACCAGGTCTTGACATC

## Data Availability

The data presented in this study are available on request from the corresponding author.
